# Efficient alignment of RNA secondary structures using sparse dynamic programming

**DOI:** 10.1186/1471-2105-14-269

**Published:** 2013-09-08

**Authors:** Cuncong Zhong, Shaojie Zhang

**Affiliations:** 1 Department of Electrical Engineering and Computer Science, University of Central Florida, Orlando, FL 32816-2362, USA

## Abstract

**Background:**

Current advances of the next-generation sequencing technology have revealed a large number of un-annotated RNA transcripts. Comparative study of the RNA structurome is an important approach to assess their biological functionalities. Due to the large sizes and abundance of the RNA transcripts, an efficient and accurate RNA structure-structure alignment algorithm is in urgent need to facilitate the comparative study. Despite the importance of the RNA secondary structure alignment problem, there are no computational tools available that provide high computational efficiency and accuracy. In this case, designing and implementing such an efficient and accurate RNA secondary structure alignment algorithm is highly desirable.

**Results:**

In this work, through incorporating the sparse dynamic programming technique, we implemented an algorithm that has an *O*(*n*^3^) expected time complexity, where *n* is the average number of base pairs in the RNA structures. This complexity, which can be shown assuming the polymer-zeta property, is confirmed by our experiments. The resulting new RNA secondary structure alignment tool is called ERA. Benchmark results indicate that ERA can significantly speedup RNA structure-structure alignments compared to other state-of-the-art RNA alignment tools, while maintaining high alignment accuracy.

**Conclusions:**

Using the sparse dynamic programming technique, we are able to develop a new RNA secondary structure alignment tool that is both efficient and accurate. We anticipate that the new alignment algorithm ERA will significantly promote comparative RNA structure studies. The program, ERA, is freely available at http://genome.ucf.edu/ERA.

## Background

Non-coding RNAs (ncRNAs) have recently been recognized as important regulators of the biological systems [1,2]. They participate in the control of alternative splicing [3], gene transcription [4] and translation [5], and mRNA localization [6]. Most of the ncRNAs exert their biological functions by folding into specific structures, which makes the study of the RNA structurome a critical step towards complete understanding of the operational mechanism of the biological system [7]. Recently, genome-wide RNA structurome analysis has led to many interesting discoveries regarding novel regulatory mechanisms. For example, analysis of the RNA structural elements in *Drosophila melanogaster* 3’-UTR suggests a cluster of ncRNA elements that can direct the localization of their upstream genes within the spermatids [8]. Similar studies have also been applied to the *Ciona intestinalis* genome for novel ncRNA family discovery [9]. With the finishing of the ENCODE [10] and modENCODE [11] projects, we expect that much more RNA transcripts will be experimentally identified. Many of these RNA transcripts may have exceptionally large sizes [12], and calls for more efficient computational tools to analyze their structures.

As more RNA transcripts are being discovered, the experimental approaches for probing ncRNA structures are also being revolutionized, allowing more accurate functional investigation through exploiting the structure-function relationship. Traditional RNA three-dimensional (3D) structure determination techniques such as X-ray crystallography, NMR and cryo-EM are expensive, making them inappropriate for genome-wide survey of RNA structures. Currently, the emerging massive parallel sequencing technology has been incorporated into the traditional chemical probing methods, making genome-wide experimental determination of RNA secondary structures possible and with low cost. Available techniques in this category include PARS [13], FragSeq [14], and SHAPE-seq [15]. The RNA secondary structures determined by these techniques are much more accurate than those predicted by pure computational methods. For example, when coupled with SHAPE-seq data, the free energy minimization approach [16] is able to predict the secondary structure of a 16S rRNA with over 95% accuracy [17]. In this case, the major purpose of this work is to develop an efficient and accurate *RNA secondary structure alignment* algorithm to facilitate genome-wide comparative studies of these RNA secondary structures.

There are many existing algorithms that focus on the RNA secondary structure alignment problem [18-24]. RNA secondary structures can be represented as tree structures, and the edit-distance between the tree structures can be used to represent their structural similarity [19]. Algorithms using such strategy are usually called *tree editing* algorithms. Using heavy path decomposition, Klein [25] improved the time complexity of the tree editing algorithm to *O*(*l*^3^*logl*). Recently, Demaine *et al.*[26] further improved the time complexity to *O*(*l*^3^) based on Klein’s algorithm. However, Jiang *et al.*[20] proposed to compute tree alignment distance for the comparison of trees. Algorithms that compute such a measure are called *tree alignment* algorithms. The tree alignment algorithm is a special case of the tree editing algorithm [27]. The tree alignment algorithm has been implemented into an RNA secondary structure alignment tool called RNAforester[21]. Both the tree editing and tree alignment algorithms rely on tree representation of the RNA structure, and make sophisticated scoring functions difficult to implement (such as the affine gap penalty for the loop regions). In addition, both tree editing and tree alignment algorithms do not treat base pairs as units of comparison, and make it difficult to implement a complete set of base-pair edit operations for RNA secondary structures editing (base-pair match, mismatch, breaking, altering, and removing; as defined by Jiang *et al.*[24]). We demonstrate such a problem by showing a real example from the implementation of the widely-used RNA secondary structure alignment tool RNAforester[21].

**Figure 1:**
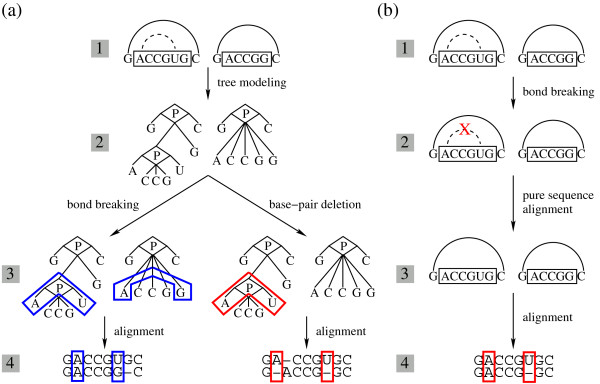
**Comparison between the tree-based alignment approach and the general edit-distance alignment approach in handling mis-predicted base pairs. (a)** The tree-based alignment algorithm in handling mis-predicted base pairs. Row 1: The arcs on the sequences indicate the base pairs (solid arc indicates real base pairs, while dashed arc indicates mis-predicted base pairs). The structure regions indicated by the boxes are being aligned. Row 2: The two RNA structures are modeled into trees according to RNAforester[21]. The ‘P’ node was introduced to represent a base pair. Row 3: Either the bond breaking or the base-pair deletion operation is taken. The blue boxes indicate the aligned nucleotides in the bond-breaking case. The red box indicates the base pair (including its nucleotides) being deleted in the base-pair deletion case. Row 4: The corresponding alignments resulted from both operations. The boxes in the alignments correspond to those in the RNA structure trees. Neither of the alignments is correct. **(b)** The general edit-distance alignment algorithm in handling mis-predicted base pairs. Row 1: The same RNA structures are being aligned. Row 2: The base-pair interaction is deleted (red cross), leaving two free nucleotides. Row 3: The sequence similarity between the boxed regions is assessed using a traditional sequence alignment algorithm [28]. Row 4: The corresponding alignment is generated correctly. The boxes correspond to nucleotides that form the mis-predicted base pair.

Consider that the two RNA structures shown in Figure 1 (a) are being aligned as trees. In the first RNA structure, due to the insertion of a uracil (U), an additional base pair is predicted (dashed arc, Row 1). Both structures are enclosed by G-C base pairs, and we focus on the alignment of their inner regions (boxed regions, Row 1). Following RNAforester’s extended tree representation [21], the two RNA structures can be transformed into two trees (Row 2). The ‘P’ node represents a base pair formed between the two corresponding nucleotides. Because there is no base pair in the second structure, the only allowed operations are bond breaking and base-pair deletion (Row 3). For the bond breaking operation, the base pair formed between A and U is broken, leaving them aligned to A and G in the second structure, respectively (blue boxes, Row 3). The alignment between the U (first structure) and G (second structure) introduces an unnecessary mismatch, making the alignment incorrect (blue boxes, Row 4). For the base-pair deletion operation, the entire base pair (including the two nucleotides A and U) is deleted (red box, Row 3). This operation opens two unnecessary gaps in the alignment (red boxes, Row 4), making it underestimate the real structural similarity. On the other hand, we expect to handle the mis-predicted base pairs in a more straightforward way. As shown in Figure 1 (b), we simply break the base pair interaction and disassociate the two corresponding nucleotides completely (red cross, Row 2). These two nucleotides are then treated as regular unpaired nucleotides. We can use the standard sequence alignment algorithm [28] (with affine gap penalty for better alignment quality in the unpaired regions) to evaluate the pure sequence similarity between the boxed hairpin-loop regions (Row 3). The resulting alignment contains only one gap, and correctly interprets the true structural difference between the two RNA structures (red boxes, Row 4).

The above example clearly shows the limitation of the implementation of the tree-based RNA secondary structure alignment algorithm RNAforester. Implementing the complete set of base-pair edit operations under the tree representation appears to be not a trivial task. Therefore, we propose to implement the general edit-distance alignment approach where all edit operations can be implemented naturally. To guarantee that the implementation is as efficient as the Demaine *et al.*’s algorithm (*O*(*l*^3^)), we incorporate the sparse DP technique into a simultaneous alignment and folding (SAF) algorithm RNAscf[29] and restrict its input to fixed RNA secondary structures (recall that the general edit-distance alignment algorithm is a restricted case of the SAF algorithm). Using this technique, we can reduce the original time complexity by reducing a factor from *n*^2^ to *z*, where *n* is the number of base pairs in the fixed RNA structures and *n*<*z*<*n*^2^. Under the assumption of the *polymer-zeta* property of RNA molecules [30], it is expected that *z*≪*n*^2^ and even *z*∈*O*(*n*). In this case, the new general edit-distance RNA structure-structure alignment algorithm will have a time complexity of *O*(*zn*^2^+*zl*^2^). The new time complexity has an expected cubic (*z*=*O*(*n*)=*O*(*l*)) growth behavior, and is the same as Demaime *et al.*’s algorithm [26]. In addition, we also devise a novel online pruning technique to further speedup the new algorithm, which deletes obsolete candidates on-the-fly. By combining both speedup techniques, the new RNA structure alignment algorithm is capable of comparing RNA secondary structures efficiently and accurately.

We have implemented the proposed RNA structure alignment algorithm into a program called ERA (Efficient RNA Alignment). The benchmark results showed that ERA has the expected *O*(*zl*^2^) time complexity. We showed the *O*(*zl*^2^) time complexity of ERA through aligning Rfam [31] RNA structures that were carefully chosen to represent a wide rage of input sizes. We also used a BraliBase II [32] benchmark to compare tools ERA, LocARNA and RNAforester when aligning RNAs with known structures. Nearly identical alignment quality can be observed for the general edit-distance alignment tools ERA and LocARNA, while both of them are more accurate than the tree alignment algorithm RNAforester. Finally, we also concluded that ERA is efficiently implemented by observing an average of 10 fold speedup over LocARNA, and RNAforester in terms of real RNA structure alignments. Based on these results, we confirmed that the sparse DP technique and the online pruning technique are successfully incorporated into the original RNAscf algorithm. We also anticipate that ERA will become an important bioinformatics tool for comparative RNA structure analysis.

## Methods

In this section, we will present a novel general edit-distance RNA structure alignment algorithm by incorporating the sparse DP technique into the RNAscf algorithm. RNAscf was originally designed to identify the consensus structure between two RNA sequences. It guides the DP process though stacks and has a time complexity of *O*(*n*^4^+*n*^2^*l*^2^). Comparing to LocARNA (which has a time complexity of *O*(*l*^4^+*n*^2^*l*^2^)), the indexing scheme used by RNAscf makes it easier to incorporate the sparse DP technique, which aims to reduce the size of *n* instead of *l*. In addition to the sparse DP technique, we will also present an online pruning technique, which tries to reduce the search space of the algorithm as the DP proceeds. Through combining these two speedup techniques, the novel algorithm will have an expected *O*(*zl*^2^) time complexity, where *n*<*z*≪*n*^2^.

The Methods section is organized as follows: In Section ‘Preliminaries and definitions’, we will give the basic definition of RNA structures and the RNA alignment problem. In Section ‘The original *O*(*n*^4^+*n*^2^*l*^2^) algorithm’, we will reintroduce the RNAscf algorithm as a basis to understand the novel algorithm that is developed in this work. In Section ‘Triangular inequality and optimal pair matchings’, we will present the triangular inequality in RNA alignment with necessary proofs, which serves as a theoretical foundation for the sparse DP technique. In Section ‘Detection of optimal pair matchings’, we will further discuss the implementation details of incorporating the sparse DP technique. In Section ‘A new algorithm with cubic time complexity’, we will present the novel RNA alignment algorithm with the incorporation of the sparse DP technique. In Section ‘Online pruning of optimal pair matchings’, we will present the online pruning technique as an additional speedup step to the novel algorithm. Finally, in Section ‘Pseudo-code’, we will summarize the new algorithm using pseudo-code that can be directly implemented.

### Preliminaries and definitions

We will begin with the introduction of the basic symbols and notations. The secondary structure of an RNA *A* of length *l*_*A*_ is represented by a set of base pairs in *A*, denoted as $P^{A}$. A base pair $p^{A}\in P^{A}$ is an interaction formed between two nucleotides in the sequence of *A*, whose positions are denoted by *l*(*p*^*A*^) and *r*(*p*^*A*^) (without loss of generality, we assume *l*(*p*^*A*^)<*r*(*p*^*A*^)). The base pair *p*^*A*^ can also be represented as (*l*(*p*^*A*^),*r*(*p*^*A*^)). The base pairs are partially ordered by the increasing order of their ending nucleotides, i.e. $p_{i}^{A}<p_{j}^{A}$ if and only if $r(p_{i}^{A})<r(p_{j}^{A})$. Since we do not consider RNA ensembles, no crossing base pair is allowed. That is, we do not allow $l(p_{i}^{A})<l(p_{j}^{A})<r(p_{i}^{A})<r(p_{j}^{A})$. The two base pairs $p_{i}^{A}$ and $p_{j}^{A}$ are either *enclosing* or *juxtaposing to* each other. The base pair $p_{j}^{A}$ encloses $p_{i}^{A}$ if $l(p_{j}^{A})<l(p_{i}^{A})<r(p_{i}^{A})<r(p_{j}^{A})$, denoted as $p_{i}^{A}{<}_{I}p_{j}^{A}$. The base pair $p_{i}^{A}$ juxtaposes to and *before*$p_{j}^{A}$ if $r(p_{i}^{A})<l(p_{j}^{A})$, and is denoted by $p_{i}^{A}{<}_{J}p_{j}^{A}$.

We also define loop regions (i.e. hairpin loop, internal/bulge loop, and multi-branch loop) whose sequence similarities are assessed by the alignment. The loop regions can be viewed as the unpaired regions in the RNA sequence that are segregated by the paired nucleotides. Let *A*[ *i*…*j*] denote a continuous sequence region in RNA *A*, which begins with the *i*th nucleotide and ends with the *j*th nucleotide. Define *L*(*p*^*A*^) as the sequence *A*[ *l*(*p*^*A*^)+1…*r*(*p*^*A*^)-1] (hairpin loop). If $p_{i}^{A}{<}_{I}p_{j}^{A}$, define $L_{l}(p_{i}^{A},p_{j}^{A})$ as the sequence $A[\;l(p_{j}^{A})+1\ldots l(p_{i}^{A})-1]$, and $L_{r}(p_{i}^{A},p_{j}^{A})$ as the sequence $A[\;r(p_{i}^{A})+1\ldots r(p_{j}^{A})-1]$ (internal or bulge loop). If $p_{i}^{A}{<}_{J}p_{j}^{A}$, define $L(p_{i}^{A},p_{j}^{A})$ as the sequence $A[\;r(p_{i}^{A})+1\ldots l(p_{j}^{A})-1]$ (multi-branch loop).

The structure alignment between RNA *A* and *B* is the optimal matching between their base-pair sets $P^{A}$ and $P^{B}$ and the corresponding loop similarities. In other words, the alignment between RNAs *A* and *B* is a one-to-one binary relation A on the base-pair sets $P^{A}$ and $P^{B}$. To ensure that the alignment will not lead to conflicting base-pair matchings, for any $(p_{i}^{A},p_{i^{\prime }}^{B})\in A$ and $(p_{j}^{A},p_{j^{\prime }}^{B})\in A$, either $p_{i}^{A}{<}_{I}p_{j}^{A}$ and $p_{i^{\prime }}^{B}{<}_{I}p_{j^{\prime }}^{B}$, or $p_{i}^{A}{<}_{J}p_{j}^{A}$ and $p_{i^{\prime }}^{B}{<}_{J}p_{j^{\prime }}^{B}$. Given the alignment  A, the matched base pairs in  A will partition the RNA sequences *A* and *B* into two sets of loop regions, ${ℒ}_{A}^{A}$ and ${ℒ}_{A}^{B}$, respectively. The sequence similarity between these two sets of loop regions is added to compute the overall alignment score. The optimal alignment is the relation  A that maximizes overall alignment score *M* that combines both structure and sequence similarities:

(1) $$M=w_{1}*\underset{(p^{A},p^{B})\in A}{\sum }S_{\text{str}}(p^{A},p^{B})+w_{2}*\sum S_{\text{seq}}({ℒ}_{A}^{A},{ℒ}_{A}^{B}).$$

Here, the first term is the summation of all structural similarities (*S*_*str*_) between the annotated base pairs. The structural similarity score for base-pair substitution is set using the RIBOSUM matrix [33], denoting such base-pair substitution matrix as *R*. We do not give penalty for base-pair deletion or insertion, as we may expect incorrectly predicted base pairs in the input RNA structures. The second term is the summation of the sequence similarities (*S*_*seq*_) on all loop (unpaired) regions that are determined by base-pair matchings in A. The sequence similarity between two sequence regions is computed as traditional sequence alignment, with *D* as a 4-by-4 matrix that accounts for nucleotide substitution (set using the RIBOSUM matrix), *g* as the gap opening penalty, and *e* as the gap extension penalty [34] (*g* and *e* are both set to negative values and *g*<*e*). The weights *w*_1_ and *w*_2_ are used to balance the structural and sequence contribution to the overall alignment score, and we set *w*_1_>*w*_2_ to emphasize structural similarity. To simplify the expressions, in the rest of this article, we assume that *w*_1_ has been multiplied to all structural similarity terms (*R*), and *w*_2_ has been multiplied to all sequence similarity terms (*D*, *g*, and *e*).

We will now define the matrices that are used by the DP algorithm. Denote *M*[ *p*^*A*^,*p*^*B*^] as the optimal structure alignment score between the regions enclosed by *p*^*A*^ and *p*^*B*^, given that *p*^*A*^ is matched with *p*^*B*^. Denote *M*_*h*_[ *p*^*A*^,*p*^*B*^] as the optimal alignment score when the matching of *p*^*A*^ and *p*^*B*^ corresponds to a hairpin loop in the consensus structure. Similarly, *M*_*l*_[ *p*^*A*^,*p*^*B*^] stores the optimal alignment score when the matching of *p*^*A*^ and *p*^*B*^ corresponds to an internal, a bulge, or a multi loop in the consensus structure. Assume that $p_{i}^{A}{<}_{I}p^{A}$, and $p_{i^{\prime }}^{B}{<}_{I}p^{B}$, *M*_*l*_[ *p*^*A*^,*p*^*B*^] can be computed by referring to the matrix $M_{c}[\;p_{i}^{A},p_{i^{\prime }}^{B}]$, which stores the optimal alignment score between the juxtaposed base-pair *chains* (each chain contains at least one base pair) that end with $p_{i}^{A}$ and $p_{i^{\prime }}^{B}$, respectively. The optimal alignment between *A* and *B* can be retrieved from $M[\;p_{0}^{A},p_{0}^{B}]$, where $p_{0}^{A}$ and $p_{0}^{B}$ are pseudo base pairs such that $p_{0}^{A}=(0,|A|-1)$, $p_{0}^{B}=(0,|B|-1)$, and $S_{\text{str}}(p_{0}^{A},p_{0}^{B})=0$[29].

### The original *O*(*n*^4^+*n*^2^*l*^2^) algorithm

In this section, we briefly reintroduce the RNAscf[29] algorithm for RNA consensus structure prediction as a basis for understanding the novel algorithm developed in this work. The recursive functions for the RNAscf algorithm are outlined as follows:

(2) $$M[\;p^{A},p^{B}]=\text{max}\left\{\begin{array}{l} M_{h}[\;p^{A},p^{B}],\\ M_{l}[\;p^{A},p^{B}]. \end{array}\right)$$

(3) $$M_{h}[\;p^{A},p^{B}]=S_{\text{str}}(p^{A},p^{B})+S_{\text{seq}}(L(p^{A}),L(p^{B})).$$

(4) $$\begin{array}{l} M_{l}[\;p^{A},p^{B}]=S_{\text{str}}(p^{A},p^{B})+\underset{i,i^{\prime }}{\text{max}}\left\{M_{c}[\;p_{i}^{A},p_{i^{\prime }}^{B}]\right)\\ \;\left(+\;S_{\text{seq}}(L_{r}(p_{i}^{A},p^{A}),L_{r}(p_{i^{\prime }}^{B},p^{B}))\right\}. \end{array}$$

(5) $$\;\begin{array}{l} M_{c}[\;p_{i}^{A},p_{i^{\prime }}^{B}]\\ \;=\;\underset{\begin{array}{l} \;p_{j}^{A}\;\in \;F(p_{i}^{A})\\ p_{j^{\prime }}^{B}\;\in \;F(p_{i^{\prime }}^{B}) \end{array}}{\text{max}}\;\left\{\;\begin{array}{l} \;M[\;p_{i}^{A},p_{i^{\prime }}^{B}]+\;S_{\text{seq}}(L_{l}(p_{i}^{A},p^{A}),L_{l}(p_{i^{\prime }}^{B},p^{B})),\\ \;M_{c}[\;p_{j}^{A},p_{j^{\prime }}^{B}]\;+\;M[\;p_{i}^{A},p_{i^{\prime }}^{B}]\;+S_{\text{seq}}(L(p_{j}^{A},p_{i}^{A}\;),L(p_{j^{\prime }}^{B},p_{i^{\prime }}^{B})\;),\\ \;M_{c}[\;p_{i}^{A},p_{j^{\prime }}^{B}]+\;G(|L(p_{j^{\prime }}^{B},p_{i^{\prime }}^{B})|+|L(p_{i^{\prime }}^{B})|),\\ \;M_{c}[\;p_{j}^{A},p_{i^{\prime }}^{B}]+\;G(|L(p_{j}^{A},p_{i}^{A})|+|L(p_{i}^{A})|). \end{array}\right) \end{array}$$

In these recursive functions, *S*_*str*_ denotes the structural similarity between two base pairs *p*^*A*^ and *p*^*B*^, *S*_*seq*_ denotes the sequence similarity between two unpaired regions, and *G* indicates the gap penalty for completely deleting the corresponding unpaired region. Note that *G*(|*L*|)=*g*+|*L*|∗*e* if |*L*|>0, and *G*(|*L*|)=0 otherwise. The base pair set $F(p_{i}^{A})$ contains all base pairs that are *directly before* and juxtaposed to $p_{i}^{A}$. In other words, if $p_{j}^{A}\in F(p_{i}^{A})$, then there is no such base pair $p_{k}^{A}$, such that $p_{j}^{A}{<}_{J}p_{k}^{A}{<}_{J}p_{i}^{A}$. In most real scenarios, |F| is considered as a constant [29,35]. This chaining technique based on the  F set enables us to handle the multi-loop case efficiently, by only considering |F| cases when computing *M*_*c*_.

Recall that the input RNA sequences have an average length of *l* and form an average of *n* base pairs. This algorithm can be computed with an expected time complexity of *O*(*n*^4^+*n*^2^*l*^2^). To see the time complexity, first note that all sequence similarity scores that are referred in the recursive functions can be computed within *O*(*n*^2^*l*^2^) time. Because all loop regions are segregated by base pairs, the number of loop regions is clearly bounded by *O*(*n*). Therefore, there are *O*(*n*^2^) combinations of loop matchings, and computing each matching requires *O*(*l*^2^) time using a standard sequence alignment algorithm [34]. To this point, we assume all sequence similarities are computed using *O*(*n*^2^*l*^2^) time, and are stored in a matrix for constant-time lookup. Now, observe that this algorithm computes the optimal alignment by filling up the DP table *M*, which contains *O*(*n*^2^) values. Computing each value in the matrix *M* depends on the corresponding values of *M*_*h*_, *M*_*l*_, and *M*_*c*_. The computation of values in matrix *M*_*h*_ can be finished in a constant time due to the pre-computed sequence similarities. The computation of *M*_*l*_ requires *O*(*n*^2^) time, as determined by the necessity of traversing all possible combinations *i* and *i*^′^ (see Equation 4). Finally, *M*_*c*_ can also be expected to be computed in a constant time, as |F| is assumed to be a constant. In this case, the computation of matrix *M* requires *O*(*n*^4^) time. Adding up the time required to pre-compute all sequence similarities of the loops, the overall time complexity for this algorithm thus becomes *O*(*n*^4^+*n*^2^*l*^2^).

### Triangular inequality and optimal pair matchings

The triangular inequality property servers as the theoretical foundation for the sparse DP technique, which saves search space while maintaining the global optimality. For computational RNA studies, this technique has been used in RNA folding [30], RNA consensus folding (SAF) [36,37], as well as RNA-RNA interaction prediction [38] applications. In this work, our aim is to bring this technique into the RNA structure alignment application, where fixed RNA structures are considered instead of RNA structure ensembles.

**Figure 2:**
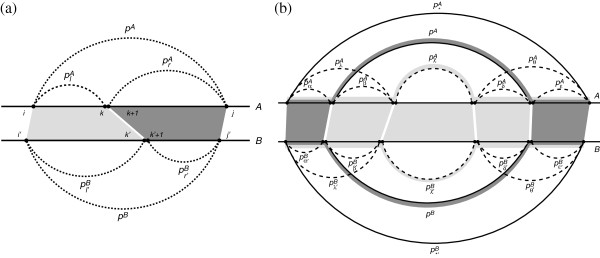
**Illustration of the triangular inequality property. (a)** Triangular inequality property of RNA secondary structure alignment. The horizontal lines indicate RNA sequences *A* and *B*. The dashed arcs are the pseudo base pairs added to the specific nucleotides, while the shaded areas define the correspondence between regions that are being aligned. **(b)** Alternative paths that go through either *p*^*A*^ and *p*^*B*^, or $p_{\chi }^{A}$ and $p_{\chi^{\prime }}^{B}$. The two shadings (dark and light gray) along the arcs represent the two alternative paths.

Consider the alignment between the RNA secondary structures within the two regions *A*[ *i*…*j*] and *B*[ *i*^′^…*j*^′^] (see Figure 2 (a)). Denote *M*[ *i*,*j*;*i*^′^,*j*^′^] as the optimal alignment score for such alignment. The triangular inequality can be summarized using the following inequality:

$$M[\;i,j;i^{\prime },j^{\prime }]\geq M[\;i,k;i^{\prime },k^{\prime }]+\;M[\;k+1,j;k^{\prime }+1,j^{\prime }],$$

where *i*≤*k*<*j* and *i*^′^≤*k*^′^<*j*^′^. This is because the partitions of the regions *A*[ *i*…*j*] and *B*[ *i*^′^…*j*^′^] at positions *k* and *k*^′^, respectively, do not necessarily compatible with the optimal alignment.

To simplify the expression of the triangular inequality property, we define a number of pseudo base pairs to indicate specific regions of interest. A pseudo base pair is a void interaction, such that the structural similarity between any two pseudo base pairs is defined to be 0. For instance, let *p* and *p*^′^ be two arbitrary pseudo base pairs, we will have *S*_*str*_(*p*,*p*^′^)=0. The pseudo base pairs are only used for the sake of representational simplicity, and are not required for the implementation of the algorithm. Define a pseudo base pair *p*^*A*^=(*i*,*j*) and a pseudo base pair *p*^*B*^=(*i*^′^,*j*^′^). In this case, the optimal alignment score between the regions *A*[ *i*…*j*] and *B*[ *i*^′^…*j*^′^], i.e. *M*[ *i*,*j*;*i*^′^,*j*^′^], can be rewritten as *M*[ *p*^*A*^,*p*^*B*^]. Similarly, define pseudo base pairs $p_{l}^{A}=(i,k)$, $p_{r}^{A}=(k+1,j)$, $p_{l^{\prime }}^{B}=(i^{\prime },k^{\prime })$, and $p_{r^{\prime }}^{B}=(k^{\prime }+1,j^{\prime })$ (see Figure 2 (a)). The triangular inequality can be simplified using the following observation:

#### Observation 1

$$M[\;p^{A},p^{B}]\geq M[\;p_{l}^{A},p_{l^{\prime }}^{B}]+\;M[\;p_{r}^{A},p_{r^{\prime }}^{B}]$$.

Using Observation 1, we can detect potential redundant computations in the original algorithm. Consider the structural configurations shown in Figure 2 (b), and assume that the base pairs *p*^*A*^ and *p*^*B*^ are being aligned at the current stage. Let $p_{*}^{A}$ and $p_{\chi }^{A}$ be arbitrary base pairs such that $p_{\chi }^{A}{<}_{I}p^{A}{<}_{I}p_{*}^{A}$. Note that $p_{\chi }^{A}$ may also represent a pseudo base pair in order to consider an arbitrary subregion enclosed by *p*^*A*^. Define pseudo base pairs $p_{\alpha }^{A}=(l(p_{*}^{A}),l(p^{A})-1)$, $p_{\beta }^{A}=(l(p^{A}),l(p_{\chi }^{A})-1)$, $p_{\delta }^{A}=(r(p_{\chi }^{A})+1,r(p^{A}))$, $p_{\varepsilon }^{A}=(r(p^{A})+1,r(p_{*}^{A}))$, $p_{\lambda }^{A}=(l(p_{*}^{A}),l(p_{\chi }^{A})-1)$, and $p_{\theta }^{A}=(r(p_{\chi }^{A})+1,r(p_{*}^{A}))$. Pseudo base pairs are also added to *B* symmetrically (see Figure 2 (b)). We can then prove Lemma 1 using Observation 1:

#### Lemma 1

If $\exists p_{\chi }^{A}$ and $p_{\chi^{\prime }}^{B}$, such that $M[\;p_{\beta }^{A},p_{\beta^{\prime }}^{B}]+\;M[p_{\chi }^{A},p_{\chi^{\prime }}^{B}]\;+\;M[p_{\delta }^{A},p_{\delta^{\prime }}^{B}]\;\geq \;M[\;p^{A},p^{B}]$, then $M[\;p_{\lambda }^{A},p_{\lambda^{\prime }}^{B}]+\;M[p_{\chi }^{A},p_{\chi^{\prime }}^{B}]\;\;+\;\;M[p_{\theta }^{A},p_{\theta^{\prime }}^{B}]\;\;\geq \;\;M[p_{\alpha }^{A},p_{\alpha^{\prime }}^{B}]\;\;+\;\;M[p^{A},p^{B}]+\;M[\;p_{\varepsilon }^{A},p_{\varepsilon^{\prime }}^{B}]$.

##### Proof

$$\begin{array}{l} M[\;p_{\lambda }^{A},p_{\lambda^{\prime }}^{B}]+\;M[\;p_{\chi }^{A},p_{\chi^{\prime }}^{B}]+\;M[\;p_{\theta }^{A},p_{\theta^{\prime }}^{B}]\\ \geq M[\;p_{\alpha }^{A},p_{\alpha^{\prime }}^{B}]+\;M[\;p_{\beta }^{A},p_{\beta^{\prime }}^{B}]\\ \;+M[\;p_{\chi }^{A},p_{\chi^{\prime }}^{B}]+\;M[\;p_{\delta }^{A},p_{\delta^{\prime }}^{B}]+\;M[\;p_{\varepsilon }^{A},p_{\varepsilon^{\prime }}^{B}]\\ \geq M[\;p_{\alpha }^{A},p_{\alpha^{\prime }}^{B}]+\;M[\;p^{A},p^{B}]+\;M[\;p_{\varepsilon }^{A},p_{\varepsilon^{\prime }}^{B}]. \end{array}$$

□

The first inequality is a direct application of Observation 1, and the second inequality is specified in the condition of Lemma 1.

#### 

Because $p_{*}^{A}$ and $p_{{*}^{\prime }}^{B}$ are arbitrary base pairs, Lemma 1 implies that the matching between *p*^*A*^ and *p*^*B*^ is guaranteed to be suboptimal. That is, the overall alignment score, given that *p*^*A*^ matches with *p*^*B*^, is always lower than that when assuming they do not match (as the matching of *p*^*A*^ and *p*^*B*^ is conflicted with the matching of $p_{\lambda }^{A}$ and $p_{\lambda^{\prime }}^{B}$, as well as the matching of $p_{\theta }^{A}$ and $p_{\theta^{\prime }}^{B}$). In this case, we can devise the DP algorithm to bypass the redundant references to the scenarios where *p*^*A*^ matches *p*^*B*^. Conversely, for the implementation of this idea, the DP algorithm will refer to the scenarios of matching *p*^*A*^ and *p*^*B*^ only when the condition specified in Lemma 1 is NOT satisfied. These necessary base-pair matchings are called the *Optimal Pair Matchings* (OPMs). If the matching of *p*^*A*^ and *p*^*B*^ is an OPM, we denote this OPM as *o*^*A*,*B*^. Similarly, we represent the OPM formed by base pairs $p_{i}^{A}$ and $p_{i^{\prime }}^{B}$ as $o_{i,i^{\prime }}^{A,B}$. The new RNA alignment algorithm will maintain an OPM list  O, which is modified online as the DP proceeds, so as to include newly identified OPMs and remove obsolete OPMs (which will be discussed in Section ‘Online pruning of optimal pair matchings’). If we assume that the RNA molecules have the *polymer-zeta* property [30], restricting the search space of the DP using the OPM list  O will reduce the time complexity of the RNA alignment algorithm to *O*(*zl*^2^) (as will be discussed in Section ‘A new algorithm with cubic time complexity’).

#### Detection of optimal pair matchings

In the previous section, we have proved that Lemma 1 can be used to detect the OPMs and save redundant computations. In this section, we will briefly discuss how it will be implemented. Lemma 1 states that if the alignment score assuming *p*^*A*^ matches *p*^*B*^ (*M*[ *p*^*A*^,*p*^*B*^]) is higher than the alignment score assuming *p*^*A*^ does not match *p*^*B*^, the matching between *p*^*A*^ and *p*^*B*^ is an OPM. Therefore, to detect the OPMs, we need to compute two alignment scores, i.e. the one when assuming *p*^*A*^ matches *p*^*B*^ and the one when assuming *p*^*A*^ does not match *p*^*B*^.

Based on previous definition, the first alignment score is computed as *M*[ *p*^*A*^,*p*^*B*^]. In this case, we only need to compute the second alignment score. However, computing the second alignment score (assuming *p*^*A*^ does not match *p*^*B*^) is difficult. Instead, we can compute the overall alignment score without assuming any restrictions. Apparently, the overall alignment score includes both cases disregarding whether *p*^*A*^ matches with *p*^*B*^. Therefore, if *M*[ *p*^*A*^,*p*^*B*^] is greater than or equal to such an overall optimal alignment, it is guaranteed to be greater than the alignment score when assuming *p*^*A*^ does not match *p*^*B*^, and ipso facto the matching of *p*^*A*^ and *p*^*B*^ is an OPM.

Recall that the alignment score *M*[ *p*^*A*^,*p*^*B*^] corresponds to the case where *p*^*A*^ matches with *p*^*B*^, and therefore it can be decomposed as the sum of two parts: the structure similarity between the two base pairs themselves *S*_*str*_(*p*^*A*^,*p*^*B*^), and the optimal alignment score between the regions *A*[ *l*(*p*^*A*^)+1…*r*(*p*^*A*^)-1] and *B*[ *l*(*p*^*B*^)+1…*r*(*p*^*B*^)-1] without any restrictions. In this case, define two pseudo base pairs ${\bar{p}}^{A}=(l(p^{A})-1,r(p^{A})+1)$ and ${\bar{p}}^{B}=(l(p^{B})-1,r(p^{B})+1)$, then $M[{\bar{p}}^{A},{\bar{p}}^{B}]$ can also be decomposed as the sum of two parts: $S_{\text{str}}({\bar{p}}^{A},{\bar{p}}^{B})$, and the optimal alignment score between the regions *A*[ *l*(*p*^*A*^)…*r*(*p*^*A*^)] and *B*[ *l*(*p*^*B*^)…*r*(*p*^*B*^)] without any restrictions. Note that ${\bar{p}}^{A}$ and ${\bar{p}}^{B}$ are both pseudo base pairs, and thus based on the definition, we have $S_{\text{str}}({\bar{p}}^{A},{\bar{p}}^{B})=0$. Therefore, $M[{\bar{p}}^{A},{\bar{p}}^{B}]$ is exactly the overall alignment score we need to detect the OPMs.

In this case, based on Lemma 1, if $M[\;p^{A},p^{B}]\geq M[\;{\bar{p}}^{A},{\bar{p}}^{B}]$, we will consider the matching of *p*^*A*^ and *p*^*B*^ as an OPM, and add the OPM *o*^*A*,*B*^ to the OPM list  O. The overhead for detecting the OPM is that we need to double the computation for each combination of *p*^*A*^ and *p*^*B*^. However, such overhead will not raise the time complexity, and it is worthy as it will lead to a more significant speedup of the algorithm. In the following section, we will devise a new algorithm by assuming that the OPM list  O is available.

#### A new algorithm with cubic time complexity

In this section, we introduce a new general edit-distance RNA structure alignment algorithm, which improves the original RNAscf algorithm based on Lemma 1 and has a time complexity of *O*(*z*(*n*^2^+*l*^2^)). Here, *z* is the size of the OPM list  O, and we expect that *z*∈*O*(*n*) when assuming *polymer-zeta* property [30]. If we also assume *O*(*n*)=*O*(*l*) (with fixed input RNA structures or efficiently pruned RNA structure ensembles), the overall time complexity of the new algorithm becomes *O*(*zl*^2^).

The new algorithm is developed based on the RNAscf algorithm [29]. Therefore, we adopt the same definition and notation as introduced in Section ‘Preliminaries and definitions’, as well as the similar recursive functions style used in Section ‘The original *O*(*n*^4^+*n*^2^*l*^2^) algorithm’. Because the computations of *M*[ *p*^*A*^,*p*^*B*^] and *M*_*h*_[ *p*^*A*^,*p*^*B*^] are boundary cases for the algorithm and are directly computed without referring to previous alignment results, the recursive functions for computing them are exactly the same as in the original algorithm:

(6) $$M[\;p^{A},p^{B}]=\text{max}\left\{\begin{array}{l} M_{h}[\;p^{A},p^{B}],\\ M_{l}[\;p^{A},p^{B}]. \end{array}\right)$$

(7) $$M_{h}[\;p^{A},p^{B}]=S_{\text{str}}(p^{A},p^{B})+S_{\text{seq}}(L(p^{A}),L(p^{B})).$$

The computation of *M*_*l*_[ *p*^*A*^,*p*^*B*^], on the other hand, refers to the previous alignment results that assumes $p_{i}^{A}$ matches $p_{i^{\prime }}^{B}$ (see Equation 4). Using Lemma 1, it is clear to see that instead of traversing all combinations of $p_{i}^{A}$ and $p_{i^{\prime }}^{B}$, we only need to consider the cases when the matching of $p_{i}^{A}$ and $p_{i^{\prime }}^{B}$ is an OPM:

(8) $$\;\begin{array}{l} M_{l}[\;p^{A},p^{B}]=S_{\text{str}}(p^{A},p^{B})\\ +\underset{\begin{array}{l} o_{i,i^{\prime }}^{A,B}\in O \end{array}}{\text{max}}\left\{M_{c}[\;p_{i}^{A},p_{i^{\prime }}^{B}]+\;S_{\text{seq}}(L_{r}(p_{i}^{A},p^{A}),L_{r}(p_{i^{\prime }}^{B},p^{B}))\right\}. \end{array}$$

Similarly, for the computation of $M_{c}[\;p_{i}^{A},p_{i^{\prime }}^{B}]$, we need to refer to the scenarios where $p_{i}^{A}$ matches $p_{i^{\prime }}^{B}$ and $p_{j}^{A}$ matches $p_{j^{\prime }}^{B}$. The matching of $p_{i}^{A}$ and $p_{i^{\prime }}^{B}$ is guaranteed to be an OPM, as ensured by Equation 8. Therefore, we only need to modify Equation 5 to ensure that the matching of $p_{j}^{A}$ and $p_{j^{\prime }}^{B}$ is an OPM:

(9) $$\begin{array}{l} M_{c}[\;p_{i}^{A},p_{i^{\prime }}^{B}]=\underset{o_{j,j^{\prime }}^{A,B}\in F(o_{i,i^{\prime }}^{A,B})}{\text{max}}\;\;\left\{\;\;\begin{array}{l} M[\;p_{i}^{A},p_{i^{\prime }}^{B}]+\;S_{\text{seq}}(L_{l}(p_{i}^{A},p^{A}),L_{l}(p_{i^{\prime }}^{B},p^{B})),\\ M_{c}[\;p_{j}^{A},p_{j^{\prime }}^{B}]+\;M[\;p_{i}^{A},p_{i^{\prime }}^{B}]+\;S_{\text{seq}}(L(p_{j}^{A},p_{i}^{A}),L(p_{j^{\prime }}^{B},p_{i^{\prime }}^{B})),\\ M_{c}[\;p_{j}^{A},p_{j^{\prime }}^{B}]+\;S_{\text{seq}}(L(p_{j}^{A},p_{i}^{A}),L(p_{j^{\prime }}^{B},p_{i^{\prime }}^{B}))+S_{\text{seq}}(L(p_{i}^{A}),L(p_{i^{\prime }}^{B})). \end{array}\right) \end{array}$$

Here, the set $F(o_{i,i^{\prime }}^{A,B})$ contains all OPMs that are directly before the OPM $o_{i,i^{\prime }}^{A,B}$. The  F set regarding the OPMs is defined as the follows. If an OPM $o_{j,j^{\prime }}^{A,B}\in F(o_{i,i^{\prime }}^{A,B})$, then either $p_{j}^{A}\in F(p_{i}^{A})$ or $p_{j^{\prime }}^{B}\in F(p_{i^{\prime }}^{B})$.

Recall that the time complexity of the original algorithm is *O*(*n*^4^+*n*^2^*l*^2^). The first term *O*(*n*^4^) results from *O*(*n*^2^) computations by traversing all combinations of *p*^*A*^ and *p*^*B*^ (see Equation 2) and *O*(*n*^2^) time for computing *M*_*l*_ (see Equation 4). In the new algorithm, we introduce the OPM constraint to Equation 8 and Equation 9, and thus reduce the time complexity for computing *M*_*l*_ from *O*(*n*^2^) to *O*(*z*). In this case, the first term *O*(*n*^4^) of the original time complexity can be reduced to *O*(*zn*^2^).

The second term *O*(*n*^2^*l*^2^) in the original time complexity results from computing the sequence similarities between all loop regions. Note that all loop similarities required for computing *M*_*l*_ (Equation 8) and *M*_*c*_ (Equation 9) are associated with OPMs. For example, in Equation 8, all the loops are defined according to $p_{i}^{A}$ and $p_{i^{\prime }}^{A}$, whose matching is expected to be an OPM. And in Equation 9, all the loops are defined according to $p_{i}^{A}$ and $p_{i^{\prime }}^{A}$, as well as $p_{j}^{A}$ and $p_{j^{\prime }}^{B}$, where both of these matchings are assumed to be OPMs. In this case, we do not need to compute loop similarities for all *O*(*n*^2^) base-pair combinations, instead we only need to compute the loop similarities that are associated with the OPMs. In this case, the time complexity for computing the sequence similarities between all loops that are required by the computation of *M*_*l*_ and *M*_*c*_ can be finished in *O*(*zl*^2^) time.

The only exception for the sequence similarity computation is the hairpin loop similarity *S*_*seq*_(*L*(*p*^*A*^),*L*(*p*^*B*^)), which is required for computing *M*_*h*_ (Equation 7). The computation of *M*_*h*_ is not constrained by the OPM list, and therefore *O*(*n*^2^*l*^2^) time is still required. To resolve this issue, we observe that most of the RNA structure alignment algorithms emphasize the structure similarity other than sequence similarity (*w*_1_>*w*_2_ in Equation 1). In this case, if there exist some base pairs within the regions enclosed by *p*^*A*^ and *p*^*B*^ to be matched, we can expect that *M*_*l*_[ *p*^*A*^,*p*^*B*^]>*M*_*h*_[ *p*^*A*^,*p*^*B*^] in Equation 6. In this case, to avoid the unnecessary computation of *M*_*h*_[ *p*^*A*^,*p*^*B*^], we can derive an upper bound ${\hat{M}}_{h}[\;p^{A},p^{B}]$, which satisfies ${\hat{M}}_{h}[\;p^{A},p^{B}]\;>\;M_{h}[\;p^{A},p^{B}]$ and can be estimated in unit time. Note that if $M_{l}[\;p^{A},p^{B}]>{\hat{M}}_{h}[\;p^{A},p^{B}]$, we are sure that *M*_*l*_[ *p*^*A*^,*p*^*B*^]>*M*_*h*_[ *p*^*A*^,*p*^*B*^] by transition, and thus can save the computation of *M*_*h*_[ *p*^*A*^,*p*^*B*^]. The upper bound ${\hat{M}}_{h}[\;p^{A},p^{B}]$ can be easily derived by assuming maximum number of nucleotide matchings and minimum number of gaps:

(10) $$\;\begin{array}{l} {\hat{M}}_{h}[\;p^{A},p^{B}]=S_{\text{str}}(p^{A},p^{B})+\min(|L(p^{A})|,|L(p^{B})|)*d_{\text{max}}\\ \;+I*g+(||L(p^{A})|-|L(p^{B})||)*e, \end{array}$$

where *d*_*max*_ is the highest score in the 4-by-4 nucleotide substitution matrix *D*, and *I* is a boolean variable that is set to 1 if |*L*(*p*^*A*^)|≠|*L*(*p*^*B*^)| and set to 0 otherwise. For the computation of each *M*[ *p*^*A*^,*p*^*B*^], we first estimate the upper bound ${\hat{M}}_{h}[\;p^{A},p^{B}]$ in a unit time, and then compute *M*_*l*_[ *p*^*A*^,*p*^*B*^] in *O*(*z*) time. By comparing these two values, we will determine whether the computation of *M*_*h*_[ *p*^*A*^,*p*^*B*^] is necessary. The computation of *M*_*h*_[ *p*^*A*^,*p*^*B*^] is only necessary when there are only a few base pair enclosed by *p*^*A*^ and *p*^*B*^ to be matched. Such condition implies the scenarios that either *p*^*A*^ or *p*^*B*^ is a real hairpin loop in the RNA structures, whose number is bounded by *O*(*n*). Overall, the hairpin loop similarity matrix *M*_*h*_ can be computed in *O*(*nl*^2^) time, and the overall time complexity of this algorithm is thus *O*(*z*(*n*^2^+*l*^2^)).

#### Online pruning of optimal pair matchings

In the previous sections, we have presented our approaches for detecting OPMs and building an OPM list  O, as well as a more efficient algorithm that is developed based on  O. Time complexity analysis of the algorithm claims that *O*(*z*(*n*^2^+*l*^2^)) time is sufficient for this new algorithm. The size of the OPM list  O, i.e. *z*, thus becomes an important factor that determines the efficiency of the novel algorithm. Under the current algorithmic setup, as well as other similar works that implement a candidate list [30,37], *z* continuously grows as the algorithm proceeds. In this case, it is desirable to devise an online pruning technique, which can remove the obsolete OPMs from  O, and thus achieve further speedup of the algorithm.

In this section, we will present such an online pruning technique to reduce the size of the OPM list  O. The intuition of this online pruning technique comes from the following observation. The RNA structures are primarily stabilized by a number of helices, or *perfectly stacked* base pairs. If $p_{j}^{A}$ is perfectly stacked on $p_{i}^{A}$, then $l(p_{j}^{A})=l(p_{i}^{A})-1$, and $r(p_{j}^{A})=r(p_{i}^{A})+1$. Consider the alignment between two helices, where each one of them contains *m*+1 perfectly stacked base pairs. Assume that the first helix contains base pairs $p_{i}^{A},p_{i+1}^{A},\ldots ,p_{i+m}^{A}$, and the second helix contains base pairs $p_{i^{\prime }}^{B},p_{i^{\prime }+1}^{B},\ldots ,p_{i^{\prime }+m}^{B}$. Based on Lemma 1, there will be at least *m* OPMs detected from such alignment, i.e. $o_{i,i^{\prime }}^{A,B},o_{i+1,i^{\prime }+1}^{A,B},\ldots ,o_{i+m,i^{\prime }+m}^{A,B}$. Apparently, maintaining all these *m* OPMs is unnecessary, as these base pairs should be aligned together as two complete helices, rather than be aligned separately as two sets of individual base pairs. In this case, maintaining only one OPM, i.e. $o_{i+m,i^{\prime }+m}^{A,B}$, is sufficient to represent such an alignment. The other *m* OPMs become obsolete as soon as the OPM $o_{i+m,i^{\prime }+m}^{A,B}$ is detected, and can be removed from the OPM list  O to improve computational efficiency. In the following paragraphs, we will extend this idea to consider all situations in addition to the perfectly stacked scenario, as well as give formal description of this technique and related proofs.

We will demonstrate the major idea of our novel online OPM pruning technique using Figure 2 (b). Imagine that at the current stage, *M*[ *p*^*A*^,*p*^*B*^] has just been computed and *o*^*A*,*B*^ has been identified as an OPM, where $o_{\chi ,\chi^{\prime }}^{A,B}$ is an arbitrary OPM that has been previously identified and is enclosed by *o*^*A*,*B*^ ($p_{\chi }^{A}{<}_{I}p^{A}$ and $p_{\chi^{\prime }}^{B}{<}_{I}p^{B}$). Our aim is to estimate whether the detection of the OPM *o*^*A*,*B*^ will make $o_{\chi ,\chi^{\prime }}^{A,B}$ obsolete. Let $p_{*}^{A}$ and $p_{{*}^{\prime }}^{B}$ be arbitrary base pairs such that $p^{A}{<}_{I}p_{*}^{A}$ and $p^{B}{<}_{I}p_{{*}^{\prime }}^{B}$. The regions enclosed by $p_{*}^{A}$ and $p_{{*}^{\prime }}^{B}$ can be partitioned using at least one of the following ways: $M[\;p_{\alpha }^{A},p_{\alpha^{\prime }}^{B}]+\;M[\;p^{A},p^{B}]+\;M[\;p_{\varepsilon }^{A},p_{\varepsilon^{\prime }}^{B}]$ (which is indicated by dark gray in Figure 2 (b)) and $M[\;p_{\lambda }^{A},p_{\lambda^{\prime }}^{B}]+\;M[\;p_{\chi }^{A},p_{\chi^{\prime }}^{B}]+\;M[\;p_{\theta }^{A},p_{\theta^{\prime }}^{B}]$ (which is indicated by light gray in Figure 2 (b)). If the corresponding score for the first path is higher than the second, $M[\;p_{\chi }^{A},p_{\chi^{\prime }}^{B}]$ will not be referred to by any future matching between arbitrary base pairs $p_{*}^{A}$ and $p_{{*}^{\prime }}^{B}$, and thus making the OPM $o_{\chi ,\chi^{\prime }}^{A,B}$obsolete. In this case, the OPM $o_{\chi ,\chi^{\prime }}^{A,B}$ can be removed from  O.

We can summarize the criterion for removing $o_{\chi ,\chi^{\prime }}^{A,B}$ as an obsolete OPM using the following inequality:

$$\begin{array}{l} M[\;p_{\alpha }^{A},p_{\alpha^{\prime }}^{B}]+\;M[\;p^{A},p^{B}]+\;M[\;p_{\varepsilon }^{A},p_{\varepsilon^{\prime }}^{B}]\geq M[\;p_{\lambda }^{A},p_{\lambda^{\prime }}^{B}]\\ \;+M[\;p_{\chi }^{A},p_{\chi^{\prime }}^{B}]+\;M[\;p_{\theta }^{A},p_{\theta^{\prime }}^{B}], \end{array}$$

which can be rewritten as:

$$\begin{array}{l} M[\;p^{A},p^{B}]-\;M[\;p_{\chi }^{A},p_{\chi^{\prime }}^{B}]\geq (M[\;p_{\lambda }^{A},p_{\lambda^{\prime }}^{B}]-\;M[\;p_{\alpha }^{A},p_{\alpha^{\prime }}^{B}])\\ \;+(M[\;p_{\theta }^{A},p_{\theta^{\prime }}^{B}]-\;M[\;p_{\varepsilon }^{A},p_{\varepsilon^{\prime }}^{B}]). \end{array}$$

To utilize such criterion, we need to have access to all values included in the above inequality. However, we only know the values at the left hand side of the inequality (*M*[ *p*^*A*^,*p*^*B*^] and $M[\;p_{\chi }^{A},p_{\chi^{\prime }}^{B}]$), while the other values at the right hand side are unknown. This is because the definitions of these pseudo base pairs are determined by $p_{*}^{A}$ and $p_{{*}^{\prime }}^{B}$, which are arbitrary base pairs that have not yet been computed by the DP algorithm. To solve this issue, observe that the score $M[\;p_{\lambda }^{A},p_{\lambda^{\prime }}^{B}]-\;M[\;p_{\alpha }^{A},p_{\alpha^{\prime }}^{B}]$ is strongly related to the regions $A[\;l(p_{\beta }^{A})\ldots r(p_{\beta }^{A})]$ and $B[\;l(p_{\beta^{\prime }}^{B})\ldots r(p_{\beta^{\prime }}^{B})]$, and $M[\;p_{\theta }^{A},p_{\theta^{\prime }}^{B}]-\;M[\;p_{\varepsilon }^{A},p_{\varepsilon^{\prime }}^{B}]$ is strongly related to the regions $A[\;l(p_{\delta }^{A})\ldots r(p_{\delta }^{A})]$ and $B[\;l(p_{\delta^{\prime }}^{B})\ldots r(p_{\delta^{\prime }}^{B})]$. Note that the regions $A[\;l(p_{\beta }^{A})\ldots r(p_{\beta }^{A})]$ and $A[\;l(p_{\delta }^{A})\ldots r(p_{\delta }^{A})]$ can be determined when *p*^*A*^ and $p_{\chi }^{A}$ are known, which makes the estimation of their impact on future alignments possible (similarly for the regions $B[\;l(p_{\beta^{\prime }}^{B})\ldots r(p_{\beta^{\prime }}^{B})]$ and $B[\;l(p_{\delta^{\prime }}^{B})\ldots r(p_{\delta^{\prime }}^{B})]$). In this case, we can develop two upper bounds ${Û}_{\beta }$ and ${Û}_{\delta }$, such that:

$$\begin{array}{l} {Û}_{\beta }\;\geq \;M[\;p_{\lambda }^{A},p_{\lambda^{\prime }}^{B}]-\;M[\;p_{\alpha }^{A},p_{\alpha^{\prime }}^{B}],\\ {Û}_{\delta }\;\geq \;M[\;p_{\theta }^{A},p_{\theta^{\prime }}^{B}]-\;M[\;p_{\varepsilon }^{A},p_{\varepsilon^{\prime }}^{B}].\; \end{array}$$

In this case, if $M[\;p^{A},p^{B}]-\;M[\;p_{\chi }^{A},p_{\chi^{\prime }}^{B}]\geq {Û}_{\beta }+{Û}_{\delta }$, we are sure that the criterion for characterizing $o_{\chi ,\chi^{\prime }}^{A,B}$ as an obsolete OPM will be satisfied, and we will be able to remove $o_{\chi ,\chi^{\prime }}^{A,B}$ from  O immediately.

Now, we can discuss the details for setting up the upper bounds ${Û}_{\beta }$ and ${Û}_{\delta }$. Because ${Û}_{\beta }$ and ${Û}_{\delta }$ are defined symmetrically, we only discuss the computation of ${Û}_{\beta }$. Note that the upper bound ${Û}_{\beta }$ needs to satisfy the condition ${Û}_{\beta }\geq M[\;p_{\lambda }^{A},p_{\lambda^{\prime }}^{B}]-\;M[\;p_{\alpha }^{A},p_{\alpha^{\prime }}^{B}]$. Clearly, the difference between $M[\;p_{\lambda }^{A},p_{\lambda^{\prime }}^{B}]-\;M[\;p_{\alpha }^{A},p_{\alpha^{\prime }}^{B}]$ directly comes from concatenating the region $A[\;l(p_{\beta }^{A})\ldots r(p_{\beta }^{A})]$ to the region $A[\;l(p_{\alpha }^{A})\ldots r(p_{\alpha }^{A})]$, as well as concatenating the region $B[\;l(p_{\beta^{\prime }}^{B})\ldots r(p_{\beta^{\prime }}^{B})]$ to the region $B[\;l(p_{\alpha^{\prime }}^{B})\ldots r(p_{\alpha^{\prime }}^{B})]$. The best case scenario for such an operation, is to assume that the concatenation of the regions $A[\;l(p_{\beta }^{A})\ldots r(p_{\beta }^{A})]$ and $B[\;l(p_{\beta^{\prime }}^{B})\ldots r(p_{\beta^{\prime }}^{B})]$ will result in as many new base-pair and nucleotide matches as possible.

Assume that there are $m_{\beta }^{A}$ base pairs that are annotated in the region $A[\;l(p_{\beta }^{A})\ldots r(p_{\beta }^{A})]$, and $m_{\beta^{\prime }}^{B}$ base pairs that are annotated in the region $B[\;l(p_{\beta^{\prime }}^{B})\ldots r(p_{\beta^{\prime }}^{B})]$. Also assume the maximum base-pair substitution score in the RIBOSUM matrix *R* is *r*_*max*_. By concatenating the regions $A[\;l(p_{\beta }^{A})\ldots r(p_{\beta }^{A})]$ and $B[\;l(p_{\beta^{\prime }}^{B})\ldots r(p_{\beta^{\prime }}^{B})]$, we introduce at most $\text{max}(\overset{A}{\underset{\beta }{m}},m_{\beta^{\prime }}^{B})$ more base-pair matchings to the alignment indicated by $M[\;p_{\alpha }^{A},p_{\alpha^{\prime }}^{B}]$. This implies the maximum structure alignment score increment of $\text{max}(\overset{A}{\underset{\beta }{m}},m_{\beta^{\prime }}^{B})*r_{\text{max}}$. Similarly, at most $\text{max}(|L(\overset{A}{\underset{\beta }{p}})|,|L(p_{\beta^{\prime }}^{B}|))$ more nucleotide matches, or gap fill-ups, are possible, compared to the existing alignment indicated by the score $M[\;p_{\alpha }^{A},p_{\alpha^{\prime }}^{B}]$. The corresponding alignment score for such case is thus: $\text{max}(|L(\overset{A}{\underset{\beta }{p}})|,|L(p_{\beta^{\prime }}^{B}|))*(d_{\text{max}}-g-e)$. To explicitly represent the upper bound using only the identified OPMs, we rename ${Û}_{\beta }$ as ${Û}_{l}[\;o_{\chi ,\chi^{\prime }}^{A,B},o^{A,B}]$ (similarly, we rename ${Û}_{\delta }$ as ${Û}_{r}[\;o_{\chi ,\chi^{\prime }}^{A,B},o^{A,B}]$). Therefore, ${Û}_{l}[\;o_{\chi ,\chi^{\prime }}^{A,B},o^{A,B}]$ and ${Û}_{r}[\;o_{\chi ,\chi^{\prime }}^{A,B},o^{A,B}]$ can be computed using the following equations:

(11) $$\;\begin{array}{l} {Û}_{l}[\;o_{\chi ,\chi^{\prime }}^{A,B},o^{A,B}]=\text{max}(\overset{A}{\underset{\beta }{m}},m_{\beta^{\prime }}^{B})*r_{\text{max}}+\text{max}(|L(\overset{A}{\underset{\beta }{p}})|,\\ \;|L(p_{\beta^{\prime }}^{B})|)*(d_{\text{max}}-g-e),\\ {Û}_{r}[\;o_{\chi ,\chi^{\prime }}^{A,B},o^{A,B}]=\text{max}(\overset{A}{\underset{\delta }{m}},m_{\delta^{\prime }}^{B})*r_{\text{max}}+\text{max}(|L(\overset{A}{\underset{\delta }{p}})|,\\ \;|L(p_{\delta^{\prime }}^{B})|)*(d_{\text{max}}-g-e). \end{array}$$

With the upper bounds ${Û}_{l}[\;o_{\chi ,\chi^{\prime }}^{A,B},o^{A,B}]$ and ${Û}_{r}[\;o_{\chi ,\chi^{\prime }}^{A,B},o^{A,B}]$, we are able to formally prove the correctness of the online OPM pruning technique:

##### Lemma 2

If $M[\;p^{A},p^{B}]-\;M[\;p_{\chi }^{A},p_{\chi^{\prime }}^{B}]\;\geq {Û}_{l}[\;o_{\chi ,\chi^{\prime }}^{A,B},o^{A,B}]+\;\;\;\;{Û}_{r}[o_{\chi ,\chi^{\prime }}^{A,B},o^{A,B}]$, where ${Û}_{l}[o_{\chi ,\chi^{\prime }}^{A,B},o^{A,B}]\;\;\;\;\geq M[p_{\lambda }^{A},p_{\lambda^{\prime }}^{B}]-\;M[\;p_{\alpha }^{A},p_{\alpha^{\prime }}^{B}]$ and ${Û}_{r}[\;o_{\chi ,\chi^{\prime }}^{A,B},o^{A,B}]\geq \;M[\;p_{\theta }^{A},p_{\theta^{\prime }}^{B}]-\;M[\;p_{\varepsilon }^{A},p_{\varepsilon^{\prime }}^{B}]$, then $M[\;p^{A},p^{B}]\;\;+\;\;M[\;p_{\alpha }^{A},p_{\alpha^{\prime }}^{B}]\;\;+\;\;M[\;p_{\varepsilon }^{A},p_{\varepsilon^{\prime }}^{B}]\;\;\geq M[\;p_{\chi }^{A},p_{\chi^{\prime }}^{B}]+\;M[\;p_{\lambda }^{A},p_{\lambda^{\prime }}^{B}]+\;M[\;p_{\theta }^{A},p_{\theta^{\prime }}^{B}]$.

###### Proof

$$\;\begin{array}{l} M[\;p^{A},p^{B}]\geq M[\;p_{\chi }^{A},p_{\chi^{\prime }}^{B}]+\;{Û}_{l}[\;o_{\chi ,\chi^{\prime }}^{A,B},o^{A,B}]+\;{Û}_{r}[\;o_{\chi ,\chi^{\prime }}^{A,B},o^{A,B}]\\ \Rightarrow M[\;p^{A},p^{B}]+\;M[\;p_{\alpha }^{A},p_{\alpha^{\prime }}^{B}]+\;M[\;p_{\varepsilon }^{A},p_{\varepsilon^{\prime }}^{B}]\\ \;\geq M[\;p_{\chi }^{A},p_{\chi^{\prime }}^{B}]+\;{Û}_{l}[\;o_{\chi ,\chi^{\prime }}^{A,B},o^{A,B}]+\;{Û}_{r}[\;o_{\chi ,\chi^{\prime }}^{A,B},o^{A,B}]\\ \;\;+M[\;p_{\alpha }^{A},p_{\alpha^{\prime }}^{B}]+\;M[\;p_{\varepsilon }^{A},p_{\varepsilon^{\prime }}^{B}]\\ \Rightarrow M[\;p^{A},p^{B}]+\;M[\;p_{\alpha }^{A},p_{\alpha^{\prime }}^{B}]+\;M[\;p_{\varepsilon }^{A},p_{\varepsilon^{\prime }}^{B}]\geq M[\;p_{\chi }^{A},p_{\chi^{\prime }}^{B}]\\ \;\;+M[\;p_{\lambda }^{A},p_{\lambda^{\prime }}^{B}]+\;M[\;p_{\theta }^{A},p_{\theta^{\prime }}^{B}]. \end{array}$$

□

As a result, when the condition given in Lemma 2 is satisfied, the enclosed OPM $o_{\chi ,\chi^{\prime }}^{A,B}$ can be readily removed.

#### Pseudo-code

**Figure 3:**
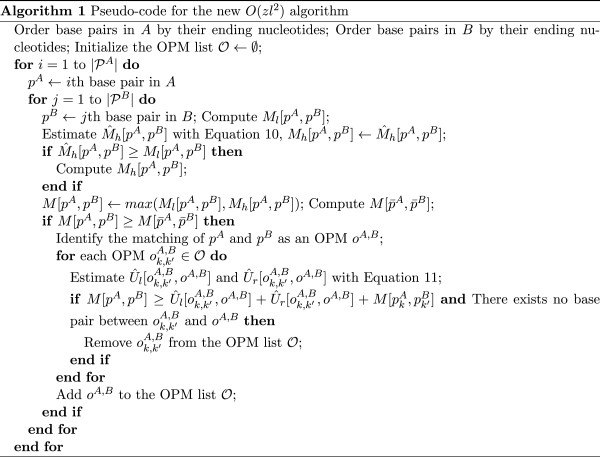
Pseudo-code for the implementation of the speedup techniques.

The pseudo-code for the new RNA secondary structure alignment algorithm that implements both speedup techniques is summarized in Figure 3.

### Results

We implemented the proposed general edit-distance RNA structural alignment algorithm into a program called ERA (Efficient RNA Alignment) using GNU C++. In this section, we will show that (1) ERA has the expected *O*(*zl*^2^) time complexity; (2) ERA is as accurate as the other state-of-the-art RNA alignment tools; and (3) ERA runs much faster than the other RNA alignment tools. In addition to these goals, we have also benchmarked ERA to demonstrate its *O*(*l*^2^) space complexity. For details regarding the space complexity issues please refer to the Additional file 1: Section S1 (also see Figure S1, Figure S2, and Table S1).

We benchmarked the ERA with two other state-of-the-art RNA alignment tools: LocARNA as a representative of the general edit-distance RNA structure alignment algorithms and RNAforester as a representative of the tree-based RNA structure alignment algorithms. Note that although LocARNA is developed to compare RNA structure ensembles, its flexible parameter setup makes it easy to prune its input RNA ensembles (see Section ‘Running LocARNA’ for more details). However, the readers should note that LocARNA is used in a restricted case for fair comparison with ERA, and more potential applications of LocARNA should be recognized. We do not compare ERA with its predecessor RNAscf, because RNAscf is implemented to find consensus helical configurations that do not include individual base pairs [29]. Both LocARNA and RNAforester were invoked using their default parameters.

#### Running LocARNA

Note that LocARNA was originally developed to compare two RNA structure ensembles [39]. Due to the recent technical advances in experimental RNA structure probing, we anticipate that RNA structures can be predicted with much higher accuracy. Therefore, we develop ERA to compare two fixed RNA structures. In this case, we need to prune the original inputs of LocARNA, so as to ensure that they only represent the fixed structures rather than any additional information.

The input RNA ensembles for LocARNA are represented using the base-pairing probability matrices, which can be computed using the McCaskill’s algorithm [40,41]. In a base-pairing probability matrix, each base pair (possibly crossing) is assigned with a probability to indicate its thermodynamic stability. Our goal is to prune such a base-pair probability matrix, such that it only contains information regarding the fixed RNA structure (in our experiment, we take the Rfam [31] annotation or the BraliBase II [32] annotation as the fixed structure for an RNA sequence). For each base pair in the matrix, if it is not presented in the annotated structure, its corresponding probability is reset to 0. On the other hand, if it is included in the annotated structure, its probability is reset to 1. In this case, the pruned base-pairing probability matrix contains only the information regarding the fixed RNA structure. We show an original and a pruned base-pairing probability matrix in Additional file 1: Figure S3 as an example. All LocARNA inputs for experiments mentioned in this article are preprocessed using this strategy.

#### Time complexity

**Figure 4:**
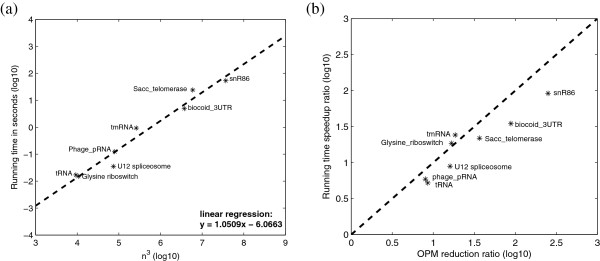
**Time complexity and OPM reduction of **ERA. **(a)** Running time versus *n*^3^, where *n* is the average number of base pairs in the RNA structures. **(b)** OPM reduction ratio versus running time speedup ratio. The OPM reduction ratio is computed by *n*^2^/*z*, where *z* is the number of OPMs.

In this section, we expect to show that the proposed sparsification is successfully implemented, and ERA has the expected *O*(*zl*^2^) time complexity. To show the *O*(*zl*^2^) time complexity, we chose a number of RNA families from Rfam that have a wide range of sequence lengths. We then randomly selected two individual RNA structures from each family (see Additional file 1: Table S2) to run ERA alignment. The running time for their alignments, versus *n*^3^ (note that *n*<*l* for annotated structures and *O*(*n*)=*O*(*l*)), is plotted in Figure 4 (a). We can clearly observe the expected *O*(*zl*^2^) time complexity from the figure. In addition, we are also able to show that the speedup ratio, when comparing to the *O*(*l*^4^+*n*^2^*l*^2^)LocARNA algorithm, is strongly correlated with the efficiency of pair matching reduction due to the sparse DP technique (the ratio *n*^2^/*z*, see Figure 4 (b)). The relatively large deviations are observed for biocoid_3UTR and snR86 RNA structures. This is because they contain a large number of base pairs and have a high base pair to sequence length ratio. In this case, the overhead for maintaining the OPM list becomes apparent and makes the speedup less significant. In summary, we have shown that the sparse DP technique is successfully implemented, ERA has an expected time complexity of *O*(*zl*^2^).

#### Alignment quality

**Figure 5:**
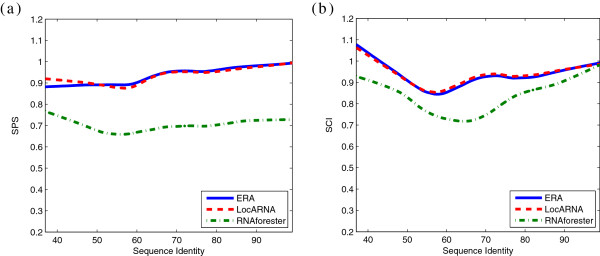
**Alignment quality comparison of **ERA, LocARNA **and **RNAforester**.** The comparison of **(a)** Sum-of-Pair Score and **(b)** Structure Conservation Index between ERA, LocARNA and RNAforester on BraliBase II data set with fixed input structures. The sequence identity range is between 0.37 to 0.99. The curves are generated using LOWESS smoothing with a smoothing factor of 0.3.

In addition to time complexity improvement, we also expect to show that ERA is as accurate as the other state-of-the-art general edit-distance RNA structure alignment tools. We used BraliBase II [32] as the reference data set, and used its corresponding structure annotations as the fixed input structures. We adopted two measures to indicate the alignment quality, i.e., the Sum-of-Pair Score (SPS) [32] and the Structure Conservation Index (SCI) [42]. The benchmark results are shown in Figure 5. The alignment qualities of ERA and LocARNA are nearly identical, since incorporating the sparse DP technique will not compromise global optimality. The benchmark results also show that ERA and LocARNA can produce more accurate alignments when compared to RNAforester. This is because ERA and the restricted version of LocARNA are both general edit-distance RNA alignment algorithms that are capable of flexibly handling incorrectly predicted base-pairs, while RNAforester is more sensitive to such errors, since it implements tree alignment.

#### Running time speedup

Finally, after benchmarking the time complexity and alignment accuracy of ERA, we also expect to show that ERA is an efficient implementation and can run faster than other state-of-the-art RNA alignment tools. We compared the real running time of ERA, LocARNA, and RNAforester on the selected RNA structures from Rfam. The benchmark results are summarized in Table 1. We can observe that ERA is capable of speeding up LocARNA by a minimum of 5.2 fold and a maximum of 91.5 fold. ERA can also speedup RNAforester by a minimum of 2.8 fold and a maximum of 242.6 fold, with only one exception in which RNAforester is 9.6 times faster than ERA. This is because the RNA structures being aligned (snR86) contain only one stem-loop structure; and in such a special case, the time complexity of RNAforester becomes *O*(*l*^2^) [21].

**Table 1:** **Comparison on running time of **ERA, LocARNA**, and **RNAforester

**RNA family**	**length**	**num.**	ERA	LocARNA	ERA **vs.**	RNAforester	ERA **vs.**
	**(bp)**	**pairs**	**(sec)**	**(sec)**	LocARNA **(fold)**	**(sec)**	RNAforester **(fold)**
tRNA	78	21	0.017	0.100	5.882	0.047	2.765
Gly riboswitch	105	22	0.015	0.277	18.46	0.162	10.80
U12 spliceosome	160	42	0.035	0.311	8.886	0.657	18.77
Phage_pRNA	244	43	0.124	0.647	5.218	6.935	55.93
tmRNA	367	64	0.929	22.45	24.16	225.4	242.6
biocoid_3UTR	549	155	4.898	170.3	34.77	13.99	2.856
snR86	1004	333	53.15	4862	91.48	5.579	-9.527 ∗
Sacc_telomerase	1162	181	23.93	522.3	21.82	3697	154.5

^*^ERA is slower than RNAforester when aligning snR86 RNA structures.

**Figure 6:**
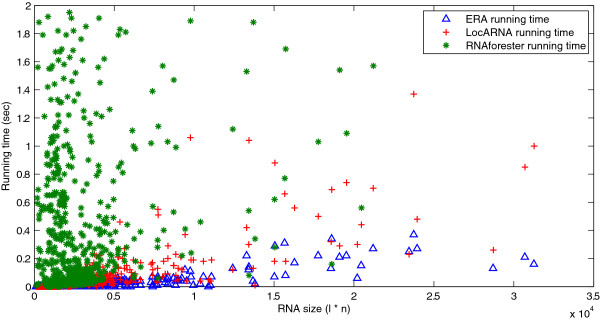
**Computational efficiency comparison between **ERA, LocARNA **and **RNAforester** on aligning randomly selected RNA structures from Rfam.** The running time for ERA (blue triangles), LocARNA (red crosses) and RNAforester (green stars) on aligning 1,000 pairs of randomly selected RNA structures from the Rfam database. The x-axis corresponds to the average sizes of the RNA structures being aligned, which is computed as the product of their average length (*l*) and their average number of base pairs (n). The y-axis corresponds to the actual running time in the unit of second. We can see that ERA is significantly faster than the other two tools.

To further investigate the real running time speedup of ERA on randomly selected RNA structures, we compiled a much larger data set that contains 1,000 pairs of randomly selected RNA structures from Rfam. The benchmark results on this large data set are summarized in Figure 6. In Figure 6, we can see that ERA (blue triangle) runs much faster than LocARNA (red cross) and RNAforester (green star). In addition, we can also observe that the running time of ERA grows slower than those of LocARNA and RNAforester, which further confirms our previous time complexity analysis (see Figure 4 (a)). This speedup is significant, and renders ERA with the power of aligning long ncRNAs that are revealed by recent research advances. In summary, ERA is an efficient and accurate RNA structure alignment tool as compared to its state-of-the-art counterparts LocARNA and RNAforester.

### Discussion and conclusions

In this article, we have presented a novel algorithm for efficient alignment of RNA secondary structures by incorporating the sparse DP technique. The major theoretical contribution of this work lies in two parts. First, to our knowledge, this is the first application of the sparse DP technique to RNA structure-structure alignment. Second, the novel online OPM pruning technique can provide insights for future algorithm designs that need to maintain a candidate list. The implementation of this novel algorithm is a tool called ERA, which can run in *O*(*zl*^2^) time and *O*(*l*^2^). Such time and space complexity make ERA one of the most efficient RNA structure alignment tools that are currently available.

The online OPM pruning technique is newly developed from this work, which aims at deleting obsolete candidates as the DP proceeds. Although this technique cannot improve the computational complexity, it is efficient in reducing the real running time. In Additional file 1: Table S3, we summarized the running time of ERA in aligning individual RNA structures, with or without the online OPM pruning technique. We observed that by incorporating this technique, the running time of ERA was reduced by an average of 2.3 fold. Meanwhile, the speedup ratio is highly uniform (with 1.7 fold as the lowest and 3.1 fold as the highest) across RNA structures with different sizes, meaning that it reduces running time by a constant factor. The online OPM pruning technique can also be modified and incorporated into other related algorithms that implement the candidate list, such as the sparse DP algorithms for RNA folding [30], RNA consensus folding [36,37], and RNA-RNA interaction [38].

The speedup of ERA is most significant when the number of base pairs in the RNA structures is small. This is because the algorithm is indexed by base pairs and has a time complexity of *O*(*z*(*n*^2^+*l*^2^)). As *n* increases, the term *O*(*zn*^2^) will dominate the overall time complexity. In this case, an ideal application of ERA is to align fixed RNA structures, because it guarantees that *n*<*l*. Note that as a sparsified version of the SAF algorithm RNAscf[29], the new algorithm developed here is also capable of handling RNA structure ensemble alignments. However, we do not implement this feature into ERA, because one cannot guarantee *n*<*l* for RNA ensemble alignments. This would make the speedup of ERA less significant. Besides, there are other alternative tools [36,37] available for such a purpose.

With the completion of the ENCODE [10] and modENCODE [11] projects, more and more RNA transcripts will be experimentally revealed. At the same time, with the advance of high-throughput RNA structure probing techniques [13-15], the secondary structures of these RNA transcripts will also be predicted with a much higher accuracy. In this case, ERA, which can compare fixed RNA structure efficiently and accurately, becomes an ideal computational tool to evaluate the structural similarities of these RNA transcripts. ERA can be used to perform all-against-all alignments on these RNA transcripts, which will then be subsequently summarized as the distance matrix for clustering purposes. Various clustering algorithms [8,39] can then be applied to identify ncRNA families with similar secondary structures and infer their amazing cellular and molecular functionalities.

### Competing interests

The authors declare that they have no competing interest.

### Authors’ contributions

SZ contributed with the conception of the research. SZ and CZ designed the algorithm. CZ implemented the algorithm and performed benchmark analysis. SZ and CZ wrote the manuscript. Both authors read and approved the final manuscript.

## Supplementary Material

Additional file 1 **Supplementary information.** This file contains four sections. In Section S1, we briefly discuss the space issue of ERA and provide related experimental results. In Section S2, we document the randomly selected RNA structures used for experiments mentioned in the main article. In Section S3, we evaluate the impact of the online OPM pruning technique in speeding up ERA. In Section S4, we give examples of the pruned base-pairing probability matrix for executing LocARNA. Click here for file
