# Supplementary material for: Efficient alignment of RNA secondary structures using sparse dynamic programming
Source: BMC Bioinformatics. 2013 Sep 8;14:269. doi: 10.1186/1471-2105-14-269 (PMC3871798; doi:10.1186/1471-2105-14-269)
Supplement: Additional file 1 — Supplementary information. This file contains four sections. In Section S1, we briefly discuss the space issue of ERA and provide related experimental results. In Section S2, we document the randomly selected RNA structures used for experiments mentioned in the main article. In Section S3, we evaluate the impact of the online OPM pruning technique in speeding up ERA. In Section S4, we give examples of the pruned base-pairing probability matrix for executing LocARNA. [file 1471-2105-14-269-S1.pdf]

Supplementary information for 'Efficient alignment of RNA secondary structures using sparse dynamic programming'

Cuncong Zhong and Shaojie Zhang*

Department of Electrical Engineering and Computer Science,
University of Central Florida, Orlando, FL, USA

S1 Space complexity of ERA

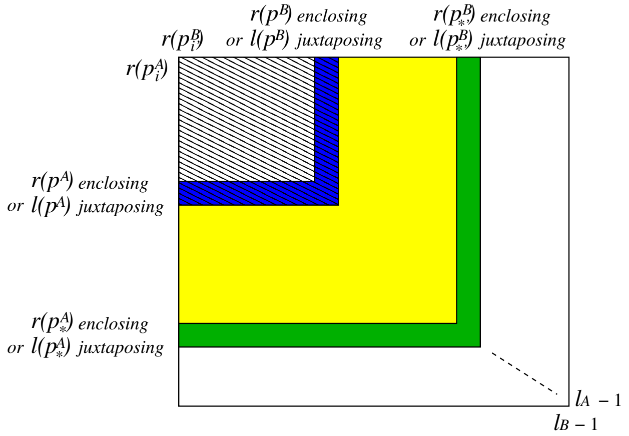

Figure S1: Schematic illustration of the technique used to achieve square space complexity. The shaded areas indicate the dynamic programming matrix that have already been filled. The blue shaded area indicates the scores on the edge that are stored, whose size is bounded by $O(l)$. The yellow and green areas indicate the sequence alignment performed, which is resumed from the scores in the blue shaded area. The green area also indicates the scores to be stored for future sequence alignments.

Here we present an implementation technique that makes the ERA algorithm computable using only $(O(max(zl, n^2)))$ space. Note that in the original algorithm, we need to store two two-dimensional matrices for each pair of p^A and p^B for constant-time lookup of loop similarities. This indicates an $O(n^2l^2)$ space complexity of the original algorithm. Although the application of OPM can reduce the space complexity to $O(zl^2)$ (we only store the two-dimensional matrices for OPMs instead of all pairs of p^A and p^B), the space complexity is still too high for long RNA structures. Therefore, we propose a more efficient space handling technique to resolve this issue.

The key idea of our space handling technique is based on the observation that the computation of each optimal sequence alignment score depends on only three other scores. If we refer to $S[i, j]$ as the optimal sequence alignment score between regions $A[0...i]$ and $B[0...j]$, then the computation of $S[i, j]$ only depends on $S[i-1, j]$, $S[i, j-1]$, and $S[i-1, j-1]$. In this case, we do not need to store the entire two-dimensional matrix, but only the scores on the 'edge' of the sequence alignment matrix are sufficient. The sequence alignments for loop regions do not need to be pre-computed, but are resumed on demand using the recorded 'edge' scores. Since the number of scores on the

*Corresponding author

edge is bounded by $O(l)$, the space for storing loop similarities can be reduced to $O(zl)$. Also note that because all sequence alignments are resumed from existing stages, no re-computation is possible. Therefore, applying this technique will not increase the time complexity.

A schematic illustration of this idea is presented in Figure S1. Let $o_{i,i'}^{A,B}$ be an OPM, as we stated before, the sequence alignment is required between regions $A[0...l(p_i^A) - 1]$, $B[0...l(p_{i'}^B) - 1]$ and between regions $A[r(p_i^A) + 1...l_A - 1]$, $B[r(p_{i'}^B) + 1...l_B - 1]$. Figure S1 shows the sequence alignment matrix for alignment between $A[r(p_i^A) + 1...l_A - 1]$, $B[r(p_{i'}^B) + 1...l_B - 1]$ (the other half adopts the same idea). The loop similarity associated with the OPM $o_{i,i'}^{A,B}$ may be demanded when there is a base pair p^A that encloses p_i^A and a base pair p^B that encloses $p_{j'}^B$ (an internal loop case), or there is another OPM $o^{A,B}$ that is juxtaposed to $o_{i,i'}^{A,B}$ (a multi-loop case). Once the loop similarity is required, we perform the sequence alignment on the corresponding regions (see detailed index in Figure S1 for enclosing or juxtaposing situations). The computed alignment results are indicated as the shaded regions. As soon as the demanded sequence similarity is returned, results stored in the shaded regions will not be requested in the future, since we do not allow crossing in RNA structures. In this case, we can discard the entire shaded regions except the scores on the edge (the blue shaded region in Figure S1). Clearly, storing the scores in the blue shaded region requires only $O(l)$ space.

As the DP proceeds, the loop similarity associated $o_{i,i'}^{A,B}$ may be requested by other base pairs or OPMs. Let the base pairs be p_*^A and $p_{*'}^B$ (enclosing) or the OPM be $o_{*,*'}^{A,B}$ (juxtaposing) (see Figure S1). To compute the corresponding sequence similarity, we can utilize the previously stored edge scores to resume the alignment. In other words, the yellow and green regions can be computed if the scores in the blue shaded region are given. After returning the requested loop similarity, the blue and yellow regions can be discarded; and the green region is stored for further references. In this case, the space for recording all loop similarities can be reduced to $O(zl)$, and the overall space complexity of this algorithm becomes $O(max(zl, n^2))$ (where n^2 is used to store matrix M and M_c).

We show the space complexity of `ERA` using RNA structures with different lengths selected from Rfam in Figure S2. The $O(l^2)$ space complexity is shown clearly. We also summarize the peak memory consumptions of `ERA` in Table S1.

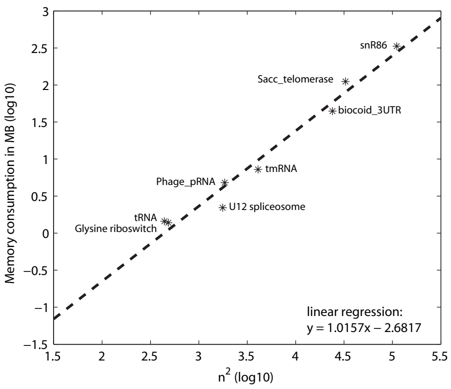

Figure S2: Space complexity of `ERA`. The peak memory consumptions (in MB) are plotted against n^2, where n is the number of base pairs and $n < l$.

Table S1: Peak memory consumptions of ERA on selected Rfam families

Name (Rfam ID)	#Pairs	Memory consumption (MB)
tRNA (RF00005)	21	1.444
Glysine riboswitch (RF00504)	22	1.380
U12 spliceosome (RF00007)	42	2.208
Phage_pRNA (RF00044)	43	4.792
tmRNA (RF00023)	64	7.248
biocoid_3UTR (RF00551)	155	44.312
snR86 (RF01272)	333	334.588
Sacc_telomerase (RF01050)	181	111.524

S2 Selected RNA structures from Rfam

The RNA families and individual structures that are selected from the Rfam database are listed as the follows.

Table S2: The individual RNA structures chosen to perform the running time experiment

Name (Rfam ID)	Structure A	Structure B
tRNA (RF00005)	AC002341.36810_6881	Z99104.295646_95728
Glysine riboswitch (RF00504)	AE000516.279183_79076	D84432.1169999_170100
U12 spliceosome (RF00007)	AAAA02006813.131506_31663	DQ888370.11_162
Phage_pRNA (RF00044)	M11813.14883_5126	V01155.1318_75
tmRNA (RF00023)	AAAK03000041.117554_17919	Z98271.113310_12943
biocoid_3UTR (RF00551)	AC002512.112119_11569	X78058.1486_1031
snR86 (RF01272)	AABY01000081.15507_6509	Z48756.113768_12765
Sacc_telomerase (RF01050)	AABY01000040.150453_51617	U14595.111_1168

S3 Speedup by the online OPM pruning technique

In the following table, we compare the running time on aligning the selected structures from Rfam, by using or without using the online OPM pruning technique. Although the online pruning technique cannot improve the time complexity, it is capable of speeding up the ERA algorithm by an average of 2.3 folds.

Table S3: The effect of the online OPM pruning technique on speeding up the ERA algorithm

Name (Rfam ID)	length (bp)	num. pairs	ERA no pruning (sec)	ERA (sec)	speedup (fold)
tRNA (RF00005)	78	21	0.036	0.017	2.118
Glysine riboswitch (RF00504)	105	22	0.030	0.015	2.000
U12 spliceosome (RF00007)	160	42	0.086	0.035	2.457
Phage_pRNA (RF00044)	244	43	0.209	0.124	1.685
tmRNA (RF00023)	367	64	1.902	0.929	2.047
biocoid_3UTR (RF00551)	549	155	15.28	4.898	3.120
snR86 (RF01272)	1004	333	130.2	53.15	2.450
Sacc_telomerase (RF01050)	1162	181	56.94	23.93	2.380

S4 Pruning base-pairing probability matrix for running LocARNA

In this section, we shown an original base-pairing probability matrix of a tmRNA, and a pruned base-pairing probability matrix of the same tmRNA in Figure S3.

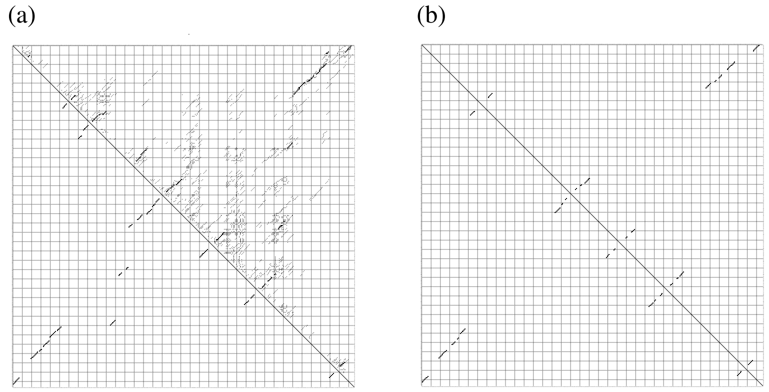

Figure S3: (a) The base-pairing probabilities of a tmRNA, which is used as `LocARNA` input when a number of structures from the ensemble are to be considered (when the minimum free energy structure is predicted with less confidence). (b) To benchmark `ERA` and `LocARNA`, we artificially remove base pairs that are not annotated. The remaining annotated base pairs are assigned with base pair probability of 1. In this case, we are able to input fixed RNA structures to `LocARNA`, and the comparison between `ERA` and `LocARNA` is fair.
